# Wegener’s granulomatosis in a middle-aged woman presenting with dyspnea, rash, hemoptysis and recurrent eye complaints: a case report

**DOI:** 10.1186/1752-1947-6-335

**Published:** 2012-10-03

**Authors:** Jose Cardenas-Garcia, Dimitrios Farmakiotis, Berta-Paola Baldovino, Peter Kim

**Affiliations:** 1 Department of Medicine, Jacobi Medical Center, Albert Einstein College of Medicine, Suite 3N1 1400 Pelham Pkwy S., Bronx, NY, 10461, USA

## Abstract

**Introduction:**

Wegener’s granulomatosis presenting as diffuse alveolar hemorrhage is uncommon. However, the recognition of multisystem disease involving joints, kidney, eye and lung is critical for diagnosing Wegener's vasculitis. This is not the first report of this kind in the literature.

**Case presentation:**

A 51-year-old Croatian woman presented to our Emergency Department with a history of progressively worsening productive cough and shortness of breath, epistaxis and two episodes of hemoptysis. She developed respiratory failure due to diffuse alveolar hemorrhage, which was successfully treated with high-dose steroids, cyclophosphamide and plasmapheresis. Her clinical course was complicated with methicillin-resistant *Staphyloccocus aureus* pneumonia, which has been associated with Wegener’s granulomatosis flares.

**Conclusion:**

The recognition of multisystem disease is critical for diagnosing Wegener's vasculitis. Diffuse alveolar hemorrhage can be a fulminant manifestation of Wegener’s granulomatosis, in which case immediate and aggressive treatment with pulse steroids, high-dose cyclophosphamide and plasma exchange can be life-saving.

## Introduction

Wegener’s granulomatosis (WG) is an antineutrophil cytoplasmic antibody (ANCA)-associated small vessel vasculitides. The clinical manifestations of vasculitides are diverse, and this is reflected in the manner of their presentation in patients in an intensive care unit (ICU). Typically, this involves the lungs or kidneys. Systemic necrotizing vasculitis represents a major challenge in critical care units, thus, early and accurate diagnosis and aggressive treatment are essential to improve outcome. The first presentation of a patient with WG to the ICU may be with respiratory failure and nonspecific changes on his or her chest radiograph rather than the more classical renal failure. We report an unusual case of WG in which the patient presented with diffuse alveolar hemorrhage (DAH), and we explore the differential diagnosis and discuss its treatment in the ICU.

## Case presentation

A 51-year-old Croatian Caucasian woman presented to our Emergency Department (ED) with a history of progressively worsening productive cough and shortness of breath, epistaxis and two episodes of hemoptysis. Over the previous 6 months, she had been seen multiple times in our ED and Medicine Clinic for nasal congestion, symptoms of upper respiratory infections and mild sinusitis, one episode of epistaxis, and she had also been referred to ophthalmology for bilateral scleral injection and serous discharge. She was subsequently diagnosed with non-necrotizing anterior scleritis 4 days prior to admission. At that time she was referred to our Medicine Clinic for a new onset of rash on her torso and legs, a painful ulcer on her lip, migratory polyarthralgias and digital swelling. Other past medical history was non-contributory and she reported no toxic habits, or use of any medication.

Because of significant hypoxia and respiratory distress, the patient was admitted to the Medical ICU. A physical examination revealed tachypnea and use of accessory muscles of respiration, bilateral conjunctival injection, prominent vessels of her nasal mucosa with a small ulceration in her right nasal cavity, few blisters and ulcers on her lower lip (consistent with herpes simplex) and one right pharyngeal pillar ulcer. Examination of the skin revealed palpable purpura on the lower abdomen and both thighs. She had bilateral symmetric arthritis of the metacarpophalangeal and proximal interphalangeal joints. On examination of her lungs there were bilateral fine crackles. The remainder of the examination was unremarkable. She was very hypoxic (PO2 50mmHg on FiO2 100%), which did not improve with bilevel positive airway pressure and she was endotracheally intubated shortly after her admission to the Medical ICU.

Microcytic anemia had developed (hemoglobin 10.4g/dL from 13.2g/dL 4 months ago), mild thrombocytosis (600,000 per microliter) was present and her erythrocyte sedimentation rate (ESR) was 100mm/hour. There was mild elevation of transaminases; blood urea nitrogen and creatinine were normal. A urine analysis revealed microscopic hematuria and significant proteinuria (>300mg/dL), without any casts.

**Figure 1:**
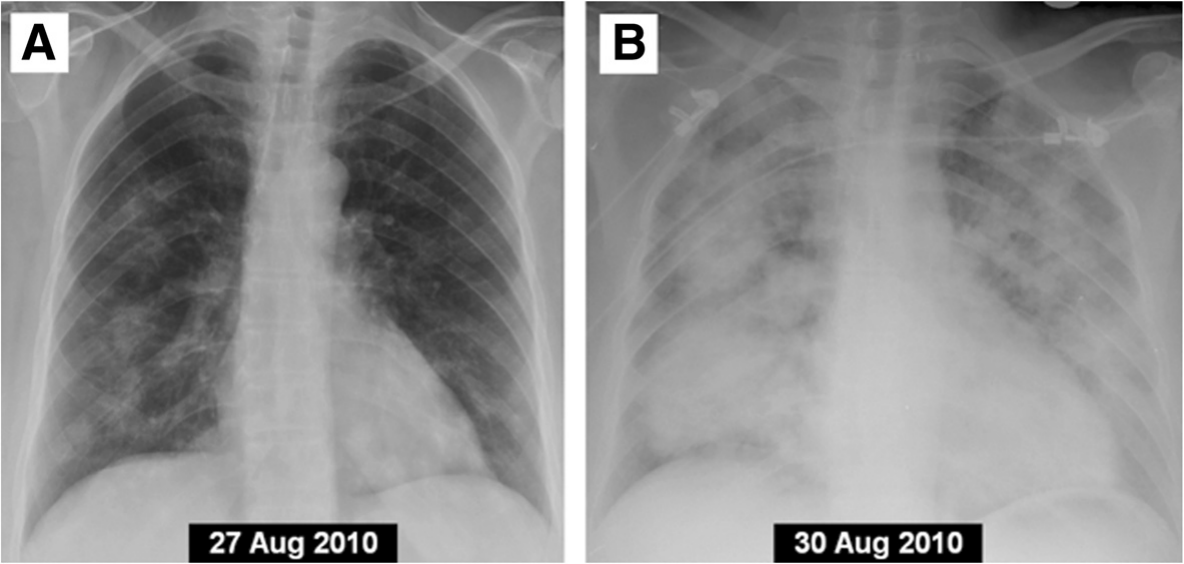
** Posterior-anterior chest X-ray 4 days prior to admission. **(**A**) Showing bilateral infiltrates. (**B**) Repeat chest X-ray on admission showing significant interval change consistent with diffuse alveolar hemorrhage.

An out-patient chest radiograph performed 4 days prior to the patient’s admission revealed bilateral alveolar infiltrates (Figure 1A). Her admission chest radiograph revealed significant interval change, consistent with DAH (Figure 1B). Her antinuclear antibody panel (ANA) and rheumatoid factor (RF) titers were negative, but her cytoplasmic (c)-ANCA autoantibodies were positive at 59U/mL. Her perinuclear (p)-ANCA and antiglomerular basement membrane (GBM) immunoglobulin titers were both negative. The result of the patient’s purified protein derivative test was negative and three acid-fast bacilli smear tests of sputum were negative. A bronchoalveolar lavage (BAL) confirmed alveolar hemorrhage. Her BAL cultures revealed methicillin-resistant *Staphylococcus aureus* (MRSA). Two sets of blood cultures drawn on admission were negative. A nasal endoscopic examination showed left synechia and whitish debris on the lateral wall and a small right ulcerated area on the right wall of her nasal septum. The nasal biopsy report described non-specific acute and chronic inflammation changes, ulceration and necrosis, but no granulomas were noted.

## Discussion

DAH is a life-threatening clinical syndrome caused by a variety of conditions, predominantly pauci-immune or immune deposit-mediated capillaritis, infections and drug reactions [1]. This patient’s progressive multisystem complaints over a period of months, along with the elevated ESR, anemia and thrombocytosis, strongly supported the diagnosis of systemic vasculitis. The differential diagnosis included WG, microscopic polyangiitis, Churg–Strauss syndrome and anti-GBM or Goodpasture syndrome (more common in men), as well as systemic lupus erythematosus and, less likely, Behçet’s disease and rheumatoid arthritis [2].

This patient’s clinical course, as well as the strongly positive c-ANCA with negative RF, ANA, p-ANCA and anti-GBM titers were considered diagnostic of WG [1,2]. Our patient had manifested many typical WG symptoms and signs, including sinusitis (51% on presentation), nasal congestion and epistaxis (36%), conjunctivitis with subsequent episcleritis (5% and 6%, respectively), frank arthritis (32%) and a purpuric rash (13%) [3].

DAH may represent an initial and rare manifestation of the disease [2,4]. Approximately 40 cases have been reported so far [2]. Usually, renal involvement is severe and the leading cause of mortality [1-3], but our patient’s renal function was preserved. However, her significant proteinuria and microscopic hematuria were also suggestive of WG-associated glomerulonephritis, despite the absence of red blood cell casts, similar to a previous report [4]. The biopsy results of her nasal cavity were non-diagnostic, most probably as a result of chronic inflammation.

DAH secondary to septic vasculitis is a known complication of infectious agents [1] but a primary bacterial, viral or fungal pneumonia alone could not have fully explained the patient’s presentation. It should be noted though that herpes simplex virus infection or reactivation as well as *Staphyloccocus aureus* carriage, both of which our patient had at the time of presentation, have been associated with WG flares [3,5].

The combination of high-dose corticosteroids and cyclophosphamide is the mainstay of treatment for the vasculitis, and disease resistance to this combination is rare. Intravenous cyclophosphamide (0.5g/m^2^ to 1.0g/m^2^ body surface area) is started at the same time as pulse methylprednisolone (1g intravenous for 3 days), followed by high-dose steroid treatment adjusted to response. Cyclophosphamide is repeated at intervals of 4 weeks. On the second day, we decided to proceed with plasma exchange (PE) because it has been used in some centers for fulminant disease causing pulmonary–renal failure requiring organ support, or within the first days following alveolar hemorrhage [6]. In our case, PE was indicated and used for five daily sessions because our patient with DAH had showed no improvement, with very high oxygen requirements on the ventilator.

Data have suggested that treatment with trimethoprim-sulfamethoxazole (TMP-SMX) may be beneficial in reducing the incidence of relapses in patients with WG in remission [7], because this antibiotic could act by eliminating the offending microbe and thereby stop the initiating stimulus [8]. TMP-SMX can be used in the initial phase of WG and some patients with less severe generalized WG [9]. TMP-SMX was prescribed on discharge for *Pneumocystis carinii* prophylaxis [10]. After a prolonged hospitalization, complicated by febrile neutropenia, MRSA bacteremia and *Pseudomonas* pneumonia, treated successfully with broad spectrum antibiotics, our patient was extubated and discharged to the Rehabilitation Unit.

## Conclusions

WG presenting as DAH is uncommon. However, the recognition of multisystem disease involving joints, kidney, eye and lung is critical for diagnosing Wegener's vasculitis. WG can progress or initially manifest as DAH, in which case immediate and aggressive treatment with pulse steroids, high-dose cyclophosphamide and PE can be life-saving.

## Consent

Written informed consent was obtained from the patient for publication of this case report and accompanying images. A copy of the written consent is available for review by the Editor-in-Chief of this journal.

## Competing interests

The authors declare that they have no competing interests.

## Authors’ contributions

BPB wrote the case presentations, JCG with DF wrote the discussion under supervision of PK. All authors read and approved the final manuscript.
